# Food Preferences in Undergraduate Nursing Students and Its Relationship with Food Addiction and Physical Activity

**DOI:** 10.3390/ijerph19073858

**Published:** 2022-03-24

**Authors:** Cristina Romero-Blanco, Antonio Hernández-Martínez, María Laura Parra-Fernández, María Dolores Onieva-Zafra, María del Carmen Prado-Laguna, Julián Rodríguez-Almagro

**Affiliations:** Department of Nursing, Physiotherapy and Occupational Therapy, Ciudad Real Faculty of Nursing, University of Castilla-La Mancha, 13071 Ciudad Real, Spain; Cristina.Romero@uclm.es (C.R.-B.); MariaLaura.Parra@uclm.es (M.L.P.-F.); MariaDolores.Onieva@uclm.es (M.D.O.-Z.); Carmina.Prado@uclm.es (M.d.C.P.-L.); JulianJ.Rodriguez@uclm.es (J.R.-A.)

**Keywords:** food, nursing students, food addiction, physical activity

## Abstract

The transition to college is a decisive stage for the acquisition of eating habits that continue into adulthood. The aim of this study is to assess the consumption of healthy elements of the Mediterranean diet in a group of university students and to evaluate whether the consumption pattern was related to sex, Body Mass Index (BMI), food addiction or the amount of physical activity performed. A total of 515 nursing students participated. The Mediterranean diet adherence questionnaire (PREDIMED), the food addiction scale (YFAS 2.0) and the International Physical Activity Questionnaire (IPAQ) were completed. For data analysis, multivariate analysis was performed with multiple linear regression and adjusted for sex, age, and BMI. The results showed that females consumed various types of meats (white/red, processed) in a healthier proportion (*p* < 0.05). Students that consumed more than one per day (unhealthy) of red/processed meats (mean difference (MD) = −0.49; 95% CI: −0.83; −0.15), soft drinks (MD = −0.82; 95% CI: 82–1.36; −0.27) and pastries (MD = −0.63; 95% CI: −0.97; −0.30) displayed higher food addiction scores. In addition, students who skipped breakfast also scored higher on food addiction (MD = 0.75; 95% CI: 0.31–1.19). Higher values of physical activity were observed in those who presented a healthy consumption of vegetables (MD = 140.86; 95% CI: 72.71–209.02), fruit (MD = 145.78; 95% CI: 69.35–222.21), legumes (MD = 136.46; 95% CI: 60.43–212.50) and nuts (MD = 74.36; 95% CI: 14.23–134.49). Students who consumed more red or processed meats, more pastries and more soft drinks had higher values of food addiction, while those who consumed more vegetables, fruits, legumes, and nuts had more minutes of physical activity per week. These findings invite us to insist on expanding knowledge regarding the health benefits of consuming a Mediterranean-type diet as a whole. The healthy consumption of fish, fruit and legumes should also be emphasized, especially among university students.

## 1. Introduction

Non-Communicable Diseases (NCDs) or chronic diseases are highly prevalent in our society. The associated mortality is equivalent to 71% of deaths worldwide, with cardiovascular diseases being the most predominant. The modifiable risk factors for these diseases include lifestyle-related aspects [1], such as physical inactivity, inadequate diet, and tobacco and alcohol consumption. The World Health Organization identified NCDs as a major obstacle for achieving the goals of the sustainable development agenda and established an action plan for the control of noncommunicable diseases [2]. The proposed strategies include dietary changes, such as increasing the consumption of fruits and vegetables, reducing salt in food, or reducing the consumption of saturated fatty acids and sugars. 

The Mediterranean diet is considered a good ally for the prevention of non-communicable diseases. Its origin is linked to the countries surrounding the Mediterranean Sea and the foods available in this environment [3]. This includes the consumption of olive oil [4], a high intake of fruits and vegetables, the consumption of whole grains and breads, nuts and fresh unprocessed foods, and a moderate consumption of other foods, such as sugars, red meat and red wine [5,6,7]. The following servings should be consumed: olive oil, four tablespoons or more per day; vegetables: two servings or more per day; fruit: three units or more per day; red or processed meats (sausages and hamburgers): less than one per day; butter: less than one per day; soft drinks: less than one per day; legumes: three or more per week; fish: three or more per week; pastries: less than two per week; nuts: more than three servings per week; white meats: preferential consumption over other meats [3].

The PREDIMED study proved the efficacy of the Mediterranean diet in the prevention of cardiovascular disease in a large cohort of subjects [8]; in addition, a greater adherence to the Mediterranean diet was associated with a lower prevalence of abdominal obesity and a lower risk of metabolic syndrome [9]. However, when evaluating the effects of the Mediterranean diet, this should not be limited to observing the consumption of a certain type of food; rather, variables associated with this consumption should also be considered, i.e., lifestyle factors and individual characteristics should be explored [3]. 

Eating habits are shaped throughout life and certain moments are key to the acquisition of a good or bad habit. The transition to university life is one of such pivotal moments as it implies a change in lifestyle that often involves the emergence of unbalanced diets or insufficient physical activity [10]. Food preferences of university students depend on multiple individual and socio-environmental factors [11], country of residence or sex determine preferences for certain foods or others [12]. Furthermore, the adherence to less healthy diets is related to other habits, such as lack of physical activity [13]. Within this population group, during their training, nursing students learn how to promote healthy lifestyles. However, they do not have better physical activity habits [14] and do not follow a Mediterranean-style diet [15], despite having acquired the necessary knowledge. These students are more prone to sedentary activities and to the consumption of ultra-processed and hyper-palatable foods, contributing to an increase in obesity [16,17]. The excessive consumption of highly palatable foods is often performed compulsively causing what some consider to be an addictive subtype of an eating disorder [18]. Although food addiction is not considered a disorder in the Diagnostic and Statistical Manual of Mental Disorders (DSM-5), its characteristics are similar to addictive disorders that are included in this manual and its evaluation is based on criteria for classifying substance dependence [19].

Considering all of the above, with this study, we set out to explore whether the food consumption of university nursing students follows the criteria of the Mediterranean diet and whether there are differences by sex or body mass index (BMI). In addition, we seek to investigate which food groups are consumed more or less healthily. Therefore, we seek to determine the relationship between food consumption and food addiction and physical activity.

## 2. Materials and Methods

### 2.1. Participants

An observational, descriptive, cross-sectional study was carried out from February to April 2021 in a sample of nursing students at the University of Castilla-La Mancha. A total of 625 nursing students were invited to participate in this study. The inclusion criteria were that students had taken the Public Health course or were enrolled in it, and all students who belonged to other Health Science disciplines other than nursing were excluded. 

#### Sample Size Calculation

To calculate the sample size, a total population of 3000 nursing students at this university was estimated.

The following parameters were used to estimate the sample: reference population of 3000 students, 95% confidence level, variance in the reference group of 3.61 points obtained through the Mediterranean Diet Adherence Questionnaire (PREDIMED) [20], accuracy of 0.20 points out of a total of 213 subjects and a loss estimate of 10%. This would require 345 study subjects.

This study is part of a project evaluating lifestyle in university students that obtained ethics committee approval under code c291 (11/19). 

### 2.2. Measurements

An online self-administered questionnaire was used to collect sociodemographic data, such as age, sex, weight, and height. These last two parameters allowed us to obtain the body mass index (BMI) of the participants using the formula BMI = weight(kg)/(height(m))^2^. The BMI variable was then divided into three categories according to the criteria of the World Health Organization (WHO), which establishes the normal range between 18.5 and 24.9 kg/m^2^. In addition, the students were asked what meals they ate each day (breakfast, lunch, lunch, snack, and dinner) and the response was dichotomized as “Yes/No”.

Evaluation of the Mediterranean diet.

The PREDIMED Mediterranean diet adherence questionnaire was used as a reference for the assessment of the Mediterranean diet [9]. This questionnaire consists of 14 questions, for each question a value is obtained, ranging from 0 to 1 point. Finally, the sum of the points obtained in the answers produces a result in terms of adherence to the Mediterranean diet. Good adherence is considered with scores of 9 points or higher, whereas <9 points is considered low adherence. For this study, the answers from this questionnaire were categorized as being “Healthy/Unhealthy” according to the 0 or 1 score of this questionnaire: if the score was zero, it was categorized as "unhealthy", and if the score was 1, it was categorized as "healthy". Following the criteria of the PREDIMED questionnaire, the following were considered healthy: olive oil: 4 tablespoons or more per day; vegetables: 2 servings or more per day; fruit: 3 units or more per day; red or processed meats (sausages and hamburgers): less than 1 per day; butter: less than 1 per day; soft drinks: less than 1 per day; legumes: 3 or more per week; fish: 3 or more per week; pastries: less than 2 per week; nuts: more than 3 servings per week; white meats: preferential consumption over other meats.

Food addiction assessment.

The Yale Food Addiction Scale (YFAS) was used, according to the latest updated version 2.0 [19,20,21]. This questionnaire consists of 35 questions with 8 response options ranging from 0 (never) to 7 (every day), which provides a symptom score ranging from 0 to 11. 

Physical activity assessment.

The International Physical Activity Questionnaire (IPAQ) short version was use2d [22]. This consists of seven questions regarding the number of days and type of physical activity performed by each individual. In this study, by analyzing the answers to this questionnaire, we were able to calculate the minutes of moderate and vigorous physical activity (MVPA) performed by the students per week, considering that the World Health Organization recommends at least 150 minutes per week [23].

### 2.3. Statistical Analysis

Descriptive statistics were performed using absolute and relative frequencies for categorical variables, and the mean and standard deviation (SD) for quantitative variables.

Subsequently, bivariate analysis was performed using Pearson’s chi-square test to determine the relationship between eating habits versus gender and BMI.

Finally, a multivariate analysis was carried out using multiple linear regression. Food addiction was assessed first as a dependent variable against all eating habits, after which physical activity was assessed. Mean differences (MD) were estimated with their respective 95% confidence intervals. In both cases, the analysis was performed unadjusted and adjusted for age, sex, and BMI. A statistical significance level of *p* < 0.05 was used.

## 3. Results

Of 625 students, a total of 515 students (82.4%) completed the questionnaire, of whom 408 were female (79.2%) with a mean age of 20.9 years SD (Table 1). A total of 411 students (80.1%) were normal weight, with the percentages of underweight or overweight/obese representing 19% of the population (49 and 53 students, respectively). The YFAS obtained a score of 0.94 (SD = 1.77) on the 11 items of the questionnaire. The mean minutes of moderate and vigorous physical activity performed weekly by the students was 236.8 (SD = 349.8), which is higher than the WHO recommendations of 150 minutes (mean 236.8 minutes). The mean score for adherence to the Mediterranean diet was 7.51 points (SD = 1.90); 70.1% (361 students) did not reach the 9 points considered for a good adherence to the Mediterranean diet. 

### 3.1. Mediterranean Diet, Gender and BMI

When food groups and meal habits were analyzed in relation to sex and BMI (Table 2), statistically significant relationships were only obtained with red/processed and white meats (*p* < 0.05). Although in both cases healthy consumption was chosen by most students, women displayed a significantly healthier consumption of both types of meat. For the remaining variables analyzed, no statistically significant differences were found. All the students responded that they ate lunch and dinner; therefore, these variables were not included in the results table. The same occurred with the variables of the PREDIMED questionnaire regarding the consumption of wine, butter, and olive oil; 100% of the sample answered homogeneously: they did not consume butter or wine, and they used olive oil for cooking.

### 3.2. Mediterranean Diet and Food Addiction

Subsequently, the analysis of dietary variables and their relationship with food addiction was performed (Table 3). Both in the unadjusted analysis and in the analysis adjusted for age, gender and BMI, statistically significant relationships were obtained with the consumption of breakfast (MD = 0.75; 95% CI: 0.31–1.19), red/processed meats (MD = −0.49; 95% CI: −0.83; −0.15), soft drinks (MD = −0.82; 95% CI: −1.36; −0.27) and pastries (MD = −0.63; 95% CI: −0.97; −0.30). Students who did not eat breakfast, and who consumed unhealthy red/processed meats, soft drinks and pastries presented significantly higher values in the food addiction score.

### 3.3. Mediterranean Diet and Physical Activity

Finally, the minutes of physical activity were assessed in relation to food consumption habits, both adjusted for age, gender, and BMI, and unadjusted (Table 4). In the unadjusted analysis, statistically significant relationships were obtained in the consumption of vegetables (MD = 140.86; 95% CI: 72.71–209.02), fruits (MD = 145.78; 95% CI: 69.35–222.21), legumes (MD = 136.46; 95% CI: 60.43–212.50) and nuts (MD = 74.36; 95% CI: 14.23–134.49). When the adjustment was made, the same relationships were observed and, in addition, pastries were added. Students with a healthy consumption of fruits, vegetables, legumes, and nuts performed more minutes of MVPA. In all cases of healthy consumption of these foods, the mean obtained was greater than 300 min per week, except for nuts (mean MVPA of 271 min). In addition, students who consumed fewer than three servings of commercial pastries per week also obtained significantly higher MVPA values than those who consumed more pastries. 

## 4. Discussion

This study analyzed the eating habits of 515 nursing students. The aim was to assess whether the food consumption pattern of the Mediterranean diet was followed in the same way by all students or whether there were differences according to sex, BMI, YFAS score or physical activity. 

The results reveal differences in meat consumption between men and women. No significant differences were seen in food consumption attributable to BMI. Those students with higher food addiction scores presented a consumption of less healthy foods, such as excess red/processed meats, soft drinks, or pastries, while consumption of healthy foods such as fruits, vegetables, legumes, and nuts were associated with high values of physical activity. No relationships were found with fish consumption or with the amount of olive oil in any of the parameters analyzed. In relation to meals, all the students ate food at lunch and dinner, while several students did not eat anything at breakfast, snack, or mid-morning lunch. Not eating breakfast was related to higher food addiction scores. 

### 4.1. Mediterranean Diet, Sex and BMI

Regarding sex, this study has shown that women tend to have a healthier consumption of meats. Sex differences in terms of meat consumption by university students have been shown in previous studies [24,25]. However, this study, unlike previous ones, enabled us to investigate the types of meat consumed, observing that women had a lower consumption of red/processed meats and a higher consumption of white meats than men. Some studies point out that the transition to university implies a change of habits towards vegetarianism among some young people, motivated by weight, and this is more frequent in women [26]. In our study, it was not possible to assess the percentage of students who were vegetarians; however, the differences obtained between men and women could also be motivated by body weight control. 

BMI was not related to any of the variables studied. The reason for this could be due to the small percentage of students who deviate from normal weight values, but also because Health Science students are more familiar with healthy eating patterns. Previous studies have shown relationships between BMI and the consumption of certain products, such as excess sugar-sweetened beverages, finding higher BMI and waist circumference values in university students [27]. In our case, there were no statistically significant relationships; moreover, the students with the lowest BMI had the least healthy consumption of soft drinks.

Regardless of gender or BMI, most students (over 80% of students) presented a healthy consumption of soft drinks and white meats, whereas healthy consumption of fruit and fish was scarce.

### 4.2. Mediterranean Diet and Food Addiction

Those students who presented worse nutritional patterns, such as skipping breakfast, eating excessive red/processed meats and soft drinks, or overconsuming pastries, presented higher values of food addiction. These findings are in line with the food preferences of people with food addiction; they are more impulsive towards the consumption of foods that are rich in sugars, fats and carbohydrates [28]. However, until now, the associations between different types of meat and food addiction had not been analyzed. A recent study evaluated the association of some types of high-fat meats with food addiction in schoolchildren and university students [29]. The results, though, were non-significant between the consumption of high-fat meat and food addiction. Nonetheless, when asked about foods that were problematic for them, in addition to including chocolate, potato chips or soft drinks, students pointed to hamburgers in the top ten [29]. In our study, those with unhealthy consumption of red/processed meats obtained higher food addiction scores. This suggests that regarding the eating pattern of people with food addition, in addition to hyperpalatable foods, other types of foods may be characteristic, such as red meat. This aspect should be further investigated since the PREDIMED questionnaire asks jointly about the consumption of red meat and processed meats. Processed meats have more fat in their composition; therefore the attraction may be due to this. It would be necessary to investigate these types of meat separately.

### 4.3. Breakfast and Food Addiction

The variable of not eating breakfast was significantly associated with higher values of food addiction. The consumption of breakfast was associated with higher BMI values [20]. In our case, no relationships were found with BMI; however we did find relationships with higher YFAS scores, which has been closely related to obesity in multiple studies, particularly in candidates for bariatric surgery [30]. Over 10% of students skip breakfast. These values are higher than others found in similar populations [31]. Therefore, the relationship between skipping breakfast and food addiction should be further investigated in future studies.

### 4.4. Mediterranean Diet and Physical Activity

The results of this study show associations between the healthy consumption of certain products and increased engagement in physical activity. Similar to these results, in eating disorders, such as orthorexia nervosa, the individual tries to eat an excessively healthy diet associated with a higher consumption of vegetables, fruits, nuts and meat [32]. In this case, students with healthy intakes of vegetables, fruit, legumes, and nuts performed more minutes of physical activity per week. The combination of healthy habits is common. Those who regularly engage in sports tend to also be concerned about their dietary habits. This is in line with previous reports in Spanish university students [33]. When assessing adherence to the Mediterranean diet, those who were more adherent to this type of diet also had higher levels of physical activity [34]. In our study, we wished to take this one step further and assess what specific elements of the Mediterranean diet were the differentiators in the performance of a greater amount of physical activity. Meat and fish were not foods that differentiated the amount of physical activity performed by the university students analyzed. Few studies have analyzed this relationship in the university population; Kim et al. [35] in a recent article observed that students who exercised three or more times per week had a higher consumption of meat, fish and eggs than those who did not exercise. The authors suggest that these findings are related to the intention to increase muscle mass through protein supplementation. In our case, significant associations were obtained with the consumption of legumes; however, total protein was not counted. The use of the PREDIMED questionnaire, which assesses the amounts and foods that improve cardiovascular health, was considered more relevant.

## 5. Limitations

This study has several limitations. The first is due to the sample used. All the participants were nursing students; therefore, we cannot predict what results would have been obtained in students with other degrees. In addition, these students are knowledgeable about nutrition and disease prevention.

Secondly, the failure to obtain results in terms of BMI could be due to the small number of overweight or obese students. It would have been very interesting to have a larger number of participants to assess whether the results were maintained. In addition, future studies could improve the anthropometric assessment by using other systems, such as bioelectrical impedance.

Finally, the questionnaires were self-administered, and therefore there could be errors in the data obtained. However, to avoid possible errors, validated questionnaires were used for the variables analyzed. 

## 6. Conclusions

This study aimed to deepen our knowledge of the diet of university students to determine whether there any foods are associated with gender, BMI, or certain eating and exercise habits. Some of the results obtained were expected, such as the data on the consumption of soft drinks, pastries, fruits and vegetables and their associations with physical activity and food addiction. However, less studied relationships have also been found, such as the different types of meat or the habit of skipping breakfast. These findings invite us to continue delving deeper into the eating patterns of this group, as well as to explore the causes that lead students to these habits. We should also insist on expanding knowledge regarding the health benefits of consuming a Mediterranean-type diet as a whole and not just limiting ourselves to compliance with certain elements. The healthy consumption of fish, fruit and legumes, for which consumption values are very low, should be emphasized, especially among university students.

## Figures and Tables

**Table 1:** Descriptive statistics of the sample analyzed.

Variables	*n* (%)	Mean (SD)
SEX		
*Women*	408 (79.2)	
*Men*	107 (20.8)	
AGE (years)		20.9 (4.01)
BMI (kg/m^2^)		22.36 (7.02)
*BMI: 18.5–24.9*	411 (80.1)	
*BMI: <18.5*	49 (9.1)	
*BMI: ≥25*	53 (9.9)	
PREDIMED SCORE		7.51 (1.90)
YFAS SCORE		0.94 (1.77)
MVPA (minutes per week)		236.80 (349.82)

MVPA: moderate and vigorous physical activity; BMI: body mass index; YFAS: Yale Food Addiction Scale.

**Table 2:** Eating habits distributed by sex and BMI.

Eating habits	SEX			BMI (kg/m^2^)			
	Women	Men	*p*	18.5–24.9	<18.5	≥25	*p*
	*n* (%)	*n* (%)	(Chi Square)	*n* (%)	*n* (%)	*n* (%)	(Chi Square)
Breakfast							
*Yes*	356 (87.3)	87 (81.3)	0.080	355 (86.4)	40 (81.6)	46 (86.8)	0.654
*No*	52 (12.7)	20 (18.7)		56 (13.6)	9 (18.4)	7 (13.2)	
Mid-morning							
*Yes*	195 (47.8)	40 (37.4)	0.054	186 (45.3)	28 (57.1)	21 (39.6)	0.182
*No*	213 (52.2)	67 (62.6)		225 (54.7)	21 (42.9)	32 (60.4)	
Mid-afternoon							
*Yes*	268 (65.7)	72 (67.3)	0.755	273 (66.4)	32 (65.3)	34 (64.2)	0.940
*No*	140 (34.3)	35 (32.7)		138 (33.6)	17 (34.7)	19 (35.8)	
Vegetables							
*Unhealthy*	298 (73.0)	85 (79.4)	0.177	303 (73.7)	39 (79.6)	39 (73.6)	0.669
*Healthy*	110 (27.0)	22 (20.6)		108 (26.3)	10 (20.4)	14 (26.4)	
Fruit							
*Unhealthy*	328 (80.4)	91 (85.0)	0.271	333 (81.0)	42 (85.7)	42 (79.2)	0.672
*Healthy*	80 (19.6)	16 (15.0)		78 (19.0)	7 (14.3)	11 (20.8)	
Red meat							
*Unhealthy*	104 (25.5)	40 (37.4)	0.015 *	117 (28.5)	15 (30.6)	11 (20.8)	0.451
*Healthy*	304 (74.5)	67 (62.6)		294 (71.5)	34 (69.4)	42 (79.2)	
Olive oil							
*Unhealthy*	188 (46.2)	57 (54.3)	0.139	195 (47.8)	23 (46.9)	26 (49.1)	0.976
*Healthy*	219 (53.8)	48 (45.7)		213 (52.2)	26 (53.1)	27 (50.9)	
Soft drinks							
*Unhealthy*	32 (7.9)	13 (12.3)	0.161	33 (8.1)	6 (12.2)	5 (9.4)	0.612
*Healthy*	372 (92.1)	93 (86.7)		373 (91.9)	43 (87.8)	48 (90.6)	
Legumes							
*Unhealthy*	333 (81.8)	84 (78.5)	0.436	340 (82.9)	37 (75.5)	38 (71.7)	0.085
*Healthy*	74 (18.2)	23 (21.5)		70 (17.1)	12 (24.5)	15 (28.3)	
Fish							
*Unhealthy*	331 (81.1)	92 (86.0)	0.243	332 (80.8)	42 (85.7)	47 (88.7)	0.289
*Healthy*	77 (18.9)	15 (14.0)		79 (19.2)	7 (14.3)	6 (11.3)	
Pastries							
*Unhealthy*	114 (28.3)	39 (36.8)	0.089	122 (30.0)	16 (32.7)	14 (27.5)	0.851
*Healthy*	289 (71.7)	67 (63.2)		285 (70.0)	33 (67.3)	37 (72.5)	
White meat							
*Unhealthy*	59 (14.5)	26 (25.0)	0.011 *	67 (16.5)	11 (22.4)	7 (13.2)	0.441
*Healthy*	347 (85.5)	78 (75.0)		339 (83.5)	38 (77.6)	46 (86.8)	
Nuts							
*Unhealthy*	194 (47.5)	47 (43.9)	0.504	195 (47.4)	20 (40.8)	25 (47.2)	0.678
*Healthy*	214 (52.5)	60 (56.1)		216 (52.6)	29 (59.2)	28 (52.8)	

* *p* < 0.05, BMI: body mass index.

**Table 3:** Food addiction and food consumption unadjusted and adjusted for sex, age, and BMI.

Eating Habits	*n* (%)	YFAS Score Mean (SD)	Coef B Value (95% CI)	Adj Coef B (95% CI)
Breakfast				
*Yes*	443(86.0)	0.84(1.59)	1 (ref.)	1 (ref.)
*No*	72 (14.0)	1.58 (2.53)	0.75 (0.31–1.18) *	0.75 (0.31–1.19) *
Mid-morning				
*Yes*	235 (45.6)	0.94 (1.73)	1 (ref.)	1 (ref.)
*No*	280 (54.4)	0.94 (1.81)	−0.01 (−0.31; −0.30)	−0.01 (−0.35; 0.269)
Mid-afternoon				
*Yes*	340 (66.0)	0.95 (1.75)	1 (ref.)	1 (ref.)
*No*	175 (34.0)	0.93 (1.81)	−0.02 (−0.34; −0.31)	−0.06 (−0.38; 0.27)
Vegetables				
*Unhealthy*	383 (74.4)	0.95 (1.71)	1 (ref.)	1 (ref.)
*Healthy*	132 (25.6)	0.92 (1.93)	−0.02 (−0.38; −0.33)	−0.03 (−0.39; 0.32)
Fruit				
*Unhealthy*	419 (81.4)	1.00 (1.79)	1 (ref.)	1 (ref.)
*Healthy*	96 (18.6)	0.69 (1.67)	−0.31 (−0.71; −0.08)	−0.30 (−069; 0.10)
Red meat				
*Unhealthy*	144 (28.0)	1.27 (1.92)	1 (ref.)	1 (ref.)
*Healthy*	371 (72.0)	0.81 (1.69)	−0.46 (−0.80; −0.12) *	−0.49 (−0.83; −0.15) *
Olive oil				
*Unhealthy*	245	0.97 (1.83)	1 (ref.)	1 (ref.)
*Healthy*	267	0.92 (1.73)	−0.05 (−0.36; 0.26)	−0.04 (−0.35; 0.28)
Soft drinks				
*Unhealthy*	45 (8.8)	1.64 (1.99)	1 (ref.)	1 (ref.)
*Healthy*	465 (91.2)	0.88 (1.74)	−0.77 (−1.31; −0.22) *	−0.82 (−1.36; −0.27) *
Legumes				
*Unhealthy*	417 (81.1)	0.91 (1.67)	1 (ref.)	1 (ref.)
*Healthy*	97 (18.9)	1.07 (2.15)	0.16 (−0.23; −0.55)	0.16 (−0.23; 0.56)
Fish				
*Unhealthy*	423 (82.1)	0.98 (1.80)	1 (ref.)	1 (ref.)
*Healthy*	92 (17.9)	0.76 (1.62)	−0.22 (−0.62; −0.18)	−0.23 (−0.63; 0.18)
Pastries				
*Unhealthy*	153 (30.1)	1.39 (2.11)	1 (ref.)	1 (ref.)
*Healthy*	356 (69.9)	0.76 (1.58)	−0.63 (−0.96; −0.30) *	−0.63 (−0.97; −0.30) *
White meat				
*Unhealthy*	85 (16.7)	1.14 (2.01)	1 (ref.)	1 (ref.)
*Healthy*	425 (83.3)	0.90 (1.70)	−0.25 (−0.65–0.17)	−0.25 (−0.66; 0.17)
Nuts				
*Unhealthy*	241 (46.8)	0.92 (1.89)	1 (ref.)	1 (ref.)
*Healthy*	274 (53.2)	0.94 (1.77)	0.05 (−0.26; −0.35)	0.06 (−0.25; 0.37)

* *p* < 0.05.

**Table 4:** Weekly physical activity and food consumption unadjusted and adjusted for sex, age and BMI.

Eating habits	*n* (%)	MVPA Mean (SD)	Coef B Value (95% CI)	Adj Coef B (95% CI)
Breakfast				
*Yes*	443(86.0)	226.13 (312.47)	1 (ref.)	1 (ref.)
*No*	72 (14.0)	302.44 (522.51)	76.32 (−10.85; 163.48)	61.45 (−25.21; 148.12)
Mid-morning				
*Yes*	235 (45.6)	220.17 (300.25)	1 (ref.)	1 (ref.)
*No*	280 (54.4)	250.75 (349.82)	30.58 (−30.23; 91.38)	21.67 (−39.05; 82.38)
Mid-afternoon				
*Yes*	340 (66.0)	251.65 (363.88)	1 (ref.)	1 (ref.)
*No*	175 (34.0)	207.95 (319.77)	−43.70 (−107.59; 20.19)	−42.22 (−105.73; 21.29)
Vegetables				
*Unhealthy*	383 (74.4)	203.95 (320.18)	1 (ref.)	1 (ref.)
*Healthy*	132 (25.6)	332.12 (410.99)	128.17 (59.64; 196.71) *	140.86 (72.71; 209.02) *
Fruit				
*Unhealthy*	419 (81.4)	211.83 (309.48)	1 (ref.)	1 (ref.)
*Healthy*	96 (18.6)	345.76 (475.32)	133.93 (56.96; 210.90) *	145.78 (69.35; 222.21) *
Red meat				
*Unhealthy*	144 (28.0)	236.63 (406.65)	1 (ref.)	1 (ref.)
*Healthy*	371 (72.0)	236.86 (325.71)	0.23 (−67.31; 67.77)	15.01 (−52.46; 82.48)
Olive oil				
*Unhealthy*	245 (47.9)	216.88 (304.31)	1 (ref.)	1 (ref.)
*Healthy*	267 (52.1)	257.02 (387.77)	40.14 (−20.75; 101.03)	50.32 (−10.23; 110.86)
Soft drinks				
*Unhealthy*	45 (8.8)	174.20 (396.79)	1 (ref.)	1 (ref.)
*Healthy*	465 (91.2)	244.06 (346.23)	69.86 (−37.77; 177.48)	82.44 (−25.24; 190.12)
Legumes				
*Unhealthy*	417 (81.1)	210.95 (324.51)	1 (ref.)	1 (ref.)
*Healthy*	97 (18.9)	350.37 (427.04)	139.42 (62.79; 216.06) *	136.46 (60.43; 212.50) *
Fish				
*Unhealthy*	423 (82.1)	231.20 (354.68)	1 (ref.)	1 (ref.)
*Healthy*	92 (17.9)	262.55 (327.16)	31.36 (−47.73; 110.45)	36.82 (−41.62; 115.26)
Pastries				
*Unhealthy*	153 (30.1)	195.95 (325.01)	1 (ref.)	1 (ref.)
*Healthy*	356 (69.9)	252.32 (355.11)	56.37 (−9.41; 122.15)	69.68 (4.22; 135.14) *
White meat				
*Unhealthy*	85 (16.7)	247.51 (475.29)	1 (ref.)	1 (ref.)
*Healthy*	425 (83.3)	236.81 (321.12)	−10.70 (−92.71; 71.31)	11.93 (−69.77; 93.62)
Nuts				
*Unhealthy*	241 (46.8)	197.19 (313.64)	1 (ref.)	1 (ref.)
*Healthy*	274 (53.2)	271.64 (375.93)	74.45 (14.04; 134.86) *	74.36 (14.23; 134.49) *

* *p* < 0.05; MVPA: moderate and vigorous physical activity (minutes per week).

## Data Availability

The data presented in this study are available on request from the corresponding author.
